# Exercise-based interventions for cancer survivors in India: a systematic review

**DOI:** 10.1186/s40064-015-1456-y

**Published:** 2015-10-31

**Authors:** Stephen R. Samuel, Sundar K. Veluswamy, Arun G. Maiya, Donald J. Fernandes, Margaret L. McNeely

**Affiliations:** Department of Physiotherapy, SOAHS, Manipal University, Manipal, India; Department of Physiotherapy, M S Ramaiah Medical College and Hospitals, MSR Nagar, MSR IT Post, Bangalore, 560054 Karnataka India; Department of Radiotherapy and Oncology, Kasturba Medical College, Manipal University, Manipal, India; Faculty of Rehabilitation Medicine, University of Alberta, Edmonton, Alberta Canada

**Keywords:** Cancer survivors, Exercise interventions, India, Research

## Abstract

Existing literature suggests that cancer survivors present with high rates of morbidity due to various treatment and disease induced factors. Research globally has shown exercise to be beneficial in improving treatment outcomes and quality of life. India has a high prevalence of cancer and not much is known about exercise interventions for cancer survivors in India. This review was planned to review the state of exercise based interventions for cancer survivors in India. A comprehensive literature search was performed in PubMed, CINAHL, EMBASE, Scopus, Cochrane Library, PEDro, IndMed, and Shoda Ganga. The search results were screened and data extracted by two independent reviewers. All eligible studies were assessed for methodological quality rating using Downs and Black checklist. Data was extracted using a pilot tested pro forma to summarize information on site and stage of cancer, type of exercise intervention and outcome measures. The review identified 13 studies, published from 1991 to 2013, after screening 4060 articles. Exercise interventions fell into one of three categories: (1) yoga-based, (2) physiotherapy-based and (3) speech therapy based interventions; and exclusively involved either breast or head and neck cancers. Studies were generally of low to moderate quality. A broad range of outcomes were found including symptoms, speech and swallowing, and quality of life and largely supported the benefits of exercise-based interventions. At present, research involving exercise-based rehabilitation interventions in India is limited in volume, quality and scope. With the growing burden of cancer in the country, there is an immediate need for research on exercise based interventions for cancer survivors within the sociocultural context of India.

## Background

India is a large low-middle income country with a significant cancer burden. Cancer incidence in India is projected to increase from 1.01 million in 2012 to 1.4 million by 2025 (Mathers et al 2012). Breast, head and neck, cervix, lung, large bowel and stomach constitute the most common sites of cancer in India and account for more than 50 % of cancer burden (Sankaranarayanan 2014). Significant advances in diagnosis and treatment of cancer has led to an increase in the proportion of cancer survivors. 10 years survival for various cancer sites in India is estimated to range from 6.4 % for cancer of oesophagus to 50.9 % for cancer of lips (Takiar et al. 2014).

As a consequence of primary treatment for cancer, survivors face a multitude of health problems and socioeconomic issues (Fairley et al. 2009). Cancer survivors frequently report adverse effects of surgery, chemotherapy and radiotherapy such as fatigue, anxiety, depression, loss of appetite, impaired joint range of motion, exercise intolerance and physical inactivity (Hewitt et al. 2003; Jones et al. 2009). These secondary complications of cancer treatment also negatively impact quality of life of cancer survivors (Osborn et al. 2006; Naughton and Weaver 2014; Curt et al. 2000). Exercise interventions involving cancer survivors have been shown to reduce cancer related fatigue, body weight and body mass index (Brown et al. 2011; Fong et al. 2012; Strasser et al. 2013); improve muscle function, body composition, peak oxygen consumption, peak power output and exercise tolerance (Jones et al. 2009; Fong et al. 2012; Strasser et al. 2013); and improve quality of life and survival (Fong et al. 2012; Mishra et al. 2012; Meyerhardt et al. 2006a, b).

Cancer research in India has grown significantly in the past two decades and its outcomes have ranged from delivering low cost, population level measures to high technology lab based outcomes (Lewison and Roe 2012; Sullivan et al. 2014). Although exercise interventions have been recognized as integral to improving outcomes of survivors of cancer from many parts of the world (Brown et al. 2011; Fong et al. 2012; Strasser et al. 2013; Mishra et al. 2012; Meyerhardt et al. 2006a, b); they do not figure in India’s cancer research priorities. Previous bibliometric analyses of cancer research in India do not describe the state of exercise related research in this population (Lewison and Roe 2012). Even reviews and commentaries on the state and future needs of palliative care in India do not identify exercise as a core component of the intervention programs (Mohanti 2011; Khosla et al. 2012). Reviewing the state of exercise related research among cancer survivors in India will help identify the state of the evidence of this essential component of cancer care in India, provide directions for future research, and could have implications for other low-middle income countries with similar cancer profile and survivorship care. This review was therefore planned to answer three specific questions: (1) What are the different cancer sites for which exercise interventions have been reported in India? (2) What are the types of exercise interventions reported from cancer survivors India? (3) What outcome measures have been reported from cancer survivors in India?

## Methods

Search strategy and selection criteria: Studies were identified by using a comprehensive search of PubMed, CINAHL, EMBASE, Scopus, Cochrane Library, PEDro, IndMed, and Shoda Ganga (a reservoir of Indian Thesis). To identify articles related to exercise interventions, we used the terms exercise; aerobic exercise; aerobic training; resistance exercise; resistance training; physical activity; flexibility training; massage; soft tissue mobilization; soft tissue manipulation; breathing exercise; breathing training; rehabilitation; prehabilitation; yoga; Tai Chi; pilates; functional training; physical therapy; physiotherapy; and speech therapy. Database specific truncation symbols were used for identifying variations of these terms. The terms cancer and neoplasm were used to identify studies related to cancer. The search was executed after combining terms related to exercise interventions, cancer and India using the Boolean operator ‘AND’. In addition, back references of selected articles were also screened manually to identify potential studies. We included randomized controlled trials (RCT), non-randomized controlled trials, single group pre-post intervention studies, case series and case reports that evaluated exercise interventions among cancer survivors in India. We limited our search to studies on humans and articles published in English language. All articles published up to the date of the search were included for the review. Primary searches were executed between October 2013–January 2014 (PubMed—23rd October 2013; CINAHL—25th November 2013; Scopus—30th November 2013; IndMed—3rd December 2013; Cochrane library and PEDro—28th December 2013; and EMBASE—January 2014). An update of the search was run on 31st March 2015 in EMBASE and 10th April 2015 in all other databases to identify publications following the primary search. The search results were screened to exclude studies on cancer survivors in intensive care units, yoga intervention that focused only on meditation, interventions addressing secondary complications of cancer like amputations, neurological impairments, prosthetic rehabilitation; and cross-sectional studies, systematic reviews and qualitative studies. Two investigators (SRS and SKV) independently executed the searches in all the databases except EMBASE. The search in EMBASE was run by the author MLM. Search results from all databases were integrated and screened independently for eligibility by SRS and SKV. Any disagreements were resolved by consensus between SRS and SKV; and if needed, through arbitration by authors AGM or DJF. Full texts of potentially eligible abstracts were obtained to assess for eligibility.

Methodological rating and data extraction: All studies eligible for the review (except case series and case reports) were independently assessed for methodological quality using the Downs and Black check list for randomised and non-randomised studies of healthcare interventions by authors SRS and SKV (Downs and Black 1998). The studies were assessed for the characteristics of reporting, external validity, internal validity-bias, internal validity-confounding, and power; for a maximum score of 32. Any disagreements were resolved by consensus between authors SRS and SKV, and if needed, through arbitration by author AGM. A data extraction form was developed, pilot tested and modified to include information regarding study objectives, site and stage of cancer, type of exercise intervention, outcome measures, study design, sample size, participant selection, medical and surgical intervention, details of exercise intervention, primary and secondary outcome measures, and adverse events as reported by the study. Data were summarised to highlight study characteristics, methodological rating score, site and stage of cancer, type of exercise interventions, and outcomes reported form each of the selected study.

## Results

**Fig. 1:**
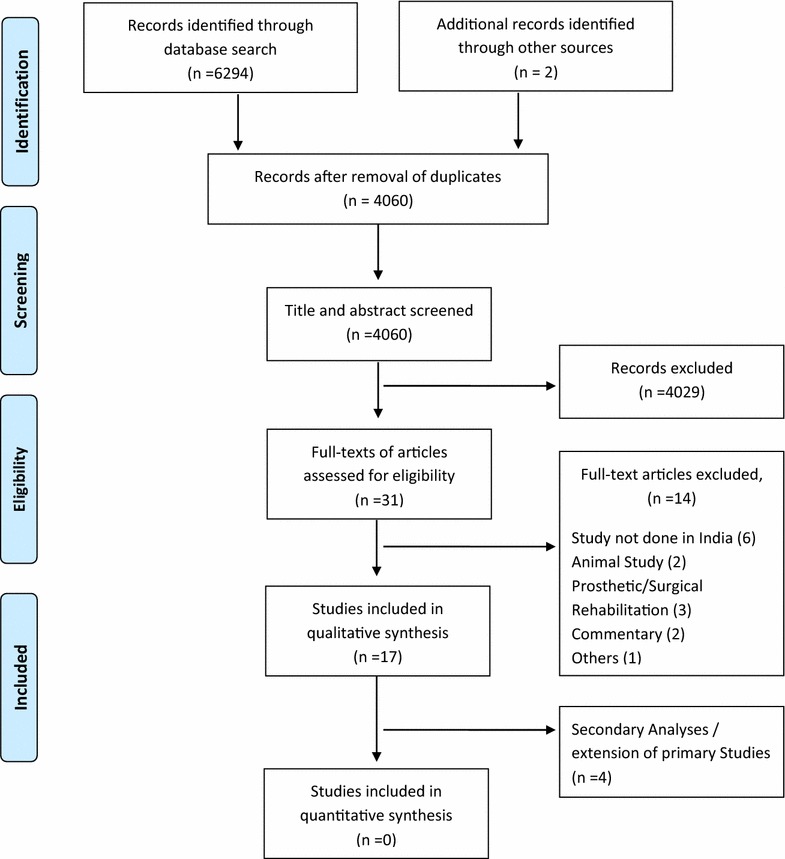
PRISMA flow diagram

A total of 6296 records were retrieved through the search strategy, of which 4060 records were screened for eligibility after excluding duplicate citations. Full texts of 31 publications were assessed for eligibility and 17 met the criteria. Six of the selected publications were based on two independent cohorts (Raghavendra et al. 2007; Rao et al. 2008a, b; Vadiraja et al. 2009a, b, c), thus resulting in 13 independent studies. The selected publications were published over a 23 year period (1991–2013) and provided data from 768 unique cancer survivors. The PRISMA flow diagram for the review is presented in Fig. 1.

Nine of the 13 studies were eligible for methodological quality assessment using the Downs and Black (1998) checklist. The scores ranged from five to 19 out of a maximum of 32 and the studies were considered to be of low to moderate methodological quality. None of the twelve studies crossed even 60 % of the maximum possible score. Only three studies were reasonably powered to assess the effect of intervention on their primary outcome measures (Gautam et al. 2011; Chakrabarty et al. 2013; Kumar et al. 2013). The total and component scores of Downs and Black checklist are summarised in Table 1.

**Table 1:** Methodological quality assessment scores using Downs and Black check list

References	Component scores of Downs and Black checklist					Maximum total score (32)
	Reporting (11)	External validity (3)	Internal validity-bias (7)	Internal validity-confounding (6)	Power (5)	
Bachher et al. (2002)	2	0	1	2	0	5
Chopra et al. (2006)	8	0	5	3	0	16
Banerjee et al. (2007)	7	0	4	3	0	14
Raghavendra et al. (2007) and Rao et al. (2008a, b)	8	0	5	4	0	17
Vadiraja et al. (2009a, b, c)	9	0	5	5	0	19
Gautam et al. (2011)	7	0	5	2	2	16
Kumar et al. (2013a, b)	7	0	4	4	1	17
Chakrabarty et al. (2013)	8	0	4	4	3	19
Samuel et al. (2013)	8	0	4	4	0	16

Among the 13 studies, the majority were RCTs (n = 6, 46 %); three (23 %) were single group pre-post intervention design; two were case series; one was a retrospective study; and one a case report. Exercise interventions were reported form survivors of predominantly two cancer sites: breast (n = 7, 53.8 %) and head and neck (n = 5, 38.5 %). In addition, one study reported its findings among a heterogeneous group of patients consisting of bone, breast, head and neck and lung cancer survivors. Three different categories of exercise interventions were identified. Among the three categories, yoga based interventions constituted 38.5 % (n = 5), and physiotherapy based and speech therapy based interventions constituted 30.8 % each (n = 4). These studies reported a diverse set of outcomes of which, quality of life was the most common (n = 5, 38.5 %) followed by stress and its markers (n = 4, 30.7 %); and anxiety and depression (n = 3, 23.1 %). Individual study characteristics including cancer sites, category of exercise intervention and outcomes reported are summarised in Table 2. The details of exercise interventions and their effects on various outcomes are summarised in Table 3. Six Publications from the two independent cohorts (Raghavendra et al. 2007; Rao et al. 2008a, b; Vadiraja et al. 2009a, b, c) reported different outcomes at different time points and have been individually summarised for greater clarity.

**Table 2:** Characteristics of all included studies

References	Cancer site; stage	Study design	Sample size	Cancer management	Exercise intervention	Outcomes assessed
Subbarao et al. (1991)	HNC (larynx); NR	Retrospective study	55	Ablative laryngeal surgery	ST	Speech proficiency
Premalatha et al. (1997)	HNC (cricoid/hypopharynx); NR	Case series	11	Laryngopharyngo-esophagectomy with gastric transposition	ST	(1) Voice quality (2) Speech intelligibility
Bachher et al. (2002)	HNC (tongue); NR	Pre-post intervention	25	2/3rd partial glossectomy	ST	(1) Speech (2) Deglutition
Chopra et al. (2006)	Breast; NR	Pre-post intervention	05	Post-operative breast cancer survivors scheduled for APBI	PT	(1) Breath hold time (2) Tidal volume
Banerjee et al. (2007)	Breast; NR	RCT	Randomised: yoga group—35; supportive therapy group—33 Completed: yoga group—35; supportive therapy group—23	RT following breast surgery	Yoga	(1) Anxiety and depression (2) Stress (3) DNA damage
Raghavendra et al. (2007)	Breast; stage II and III	RCT	Randomised: yoga group—49; supportive therapy group—49 Completed: yoga group—28; supportive therapy group—34	Adjuvant CT	Yoga	(1) Nausea and vomiting (2) Anxiety (3) Depression (4) QoL
Rao et al. (2008a, b)	Breast; stage II and III	RCT	Randomised: yoga group—49; supportive therapy group—49 Completed: yoga group—33; supportive counselling group—36	Breast surgery	Yoga	(1) Duration of drain retention, postoperative stay, hospital stay, suture removal (2) Postoperative complications (3) Cytokine levels (4) Anxiety, depression, distress and QoL (5) Immune markers
Vadiraja et al. (2009a ,b, c)	Breast; stage II and III	RCT	Randomised: yoga group—44; supportive counselling group—44. Completed: yoga group—42; supportive counselling group—33	Breast surgery followed by RT	Yoga	(1) Mood and distress (2) QoL (3) Anxiety and depression (4) Stress
Gautam et al. (2011)	Breast; stage I, II	Pre-post intervention	Enrolled—38; Completed—32	Unilateral mastectomy, CRT	PT	(1) Limb circumference and volume (2) QoL
John et al. (2011)	HNC (Buccal mucosa); stage IV	Case report	One	Composite resection of Buccal mucosa treated with PMMC flap	ST	(1) Swallowing (2) Speech Quality
Kumar et al. (2013)	Breast; stage IIb, III, IV	RCT	Randomised: intervention group—78; control group—69 Completed—intervention group—57; control group—58	RT, CT and surgery	Yoga	(1) Blood Cortisol level (2) Pain perception
Kumar et al. (2013)	Bone, breast, HNC, lung; NR	Case series	24	Analgesics for pain management as outlined by WHO analgesic ladder	PT	(1) Pain (2) QoL
Chakrabarty et al. (2013)	Breast; stage I, II and III	RCT	Randomised: yoga group—80; control group—80 completed: yoga group—80; control group—80	Surgery, CT followed by RT	Yoga	Anti-oxidants
Samuel et al. (2013)	HNC; NR	RCT	Randomised: exercise group—24; control group—24 Completed: exercise group—20; control group—23	CRT	PT	(1) Functional capacity (2) QoL

*APBI* accelerated partial breast irradiation, *CT* chemotherapy, *CRT* chemoradiotherapy, *HNC* head and neck cancer, *NR* not reported, *PMMC* pectoralis major myocutaneous flap, *PT* physiotherapy, *QoL* quality of life, *RCT* randomised controlled trial, *RT* radiotherapy, *ST* speech therapy, *WHO* World Health Organization

**Table 3:** Details of exercise interventions and their effect of reported outcomes

References	Exercise intervention	Results				
*Yoga based interventions*						
Banerjee et al. (2007)	*Intervention group* 18–24 supervised yoga sessions for 6 weeks consisting of slow stretching, asanas and breathing exercises *Control group* 3–4 supportive counselling sessions in 6 weeks and light exercises	*HADS—anxiety scores* ^a^ Yoga group: ↓48.2 %; control group: ↑28 % *HADS—depression scores* ^a^ Yoga group: ↓57.5 %; control group: ↑24 % *Perceived stress scale* ^a^ Yoga group: ↓20.4 %; control group: no change *DNA damage* ^a^ Yoga group: ↑934.6 %; Control group: ↑1028.5 % *Adverse events* none				
Raghavendra et al. (2007)	*Intervention group* Post-CT bedside yoga relaxation for 30 min; home based yoga module aided by audio and video cassettes; supervised sessions once in 10 days at home by a trainer *Control group* 30–60 min supporting counselling (information and coping preparation regarding CINV, food aversions and nutrition) during hospital visits for CT	*MANE scores*	Yoga group		Control group	
		Post CT nausea frequency^b^	3.6 ± 1.6		4.5 ± 0.9	
		Post CT nausea intensity^b^	2.3 ± 1.2		3.4 ± 1.1	
		Anticipatory nausea intensity^b^	0.6 ± 1.03		1.7 ± 1.5	
		Anticipatory vomiting intensity^c^	0.3 ± 0.67		0.87 ± 1.3	
		*STAI* ^a^	29.2 ± 3.8		37.5 ± 7.6	
		*BDI*	6.6 ± 4.6		14.2 ± 6.6	
		*FLIC* ^a^	142.1 ± 10.2		111.7 ± 25.5	
		*Adverse events* none				
Rao et al. (2008a)	*Intervention group* Instructor administered pranayama and yogic relaxation techniques at bed-side prior to surgery and during post-operative period *Control group* Four in-person supportive counselling and shoulder exercise sessions		Yoga group		Control group	
		Days drain retained^a^	4.7 ± 1.6		6.4 ± 2.5	
		Postop duration (days)	21.7 ± 9.4		24.6 ± 10.9	
		Suture removal interval (days)^c^	10.3 ± 3.6		12.7 ± 5.2	
		Postop complication^c^	6.1 %		22.2 %	
		*TNF-α levels* ↓27.3 %; ↑40.5 % (pg/ml)^a^ *Adverse events* NR				
Rao et al. (2008b)	*Intervention group* Instructor administered pranayama and yogic relaxation techniques at bed-side prior to surgery and during post-operative period; followed by 4-week home based program monitored telephonically once a week and aided by audio taped instruction *Control group* Four in-person supportive counselling and shoulder exercise sessions and home program	*STAI-anxiety state score* ^b^ Yoga group: ↓23 %; Control group: ↓22.6 % *STAI-anxiety state score* ^b^ Yoga group: ↓22 %; Control group: ↓15 % *BDI* ^b^ Yoga group: ↓4 %; Control group: No change *FLCI* ^b^ Yoga group: ↓2 %; Control group: ↓8 % *IgG* ^c^ Yoga group: ↑5.3 %; Control group: ↑43.1 % *IgA* ^b^ Yoga group: ↑2.7 %; Control group: ↑53.3 % *IgM* Yoga group: ↑12.5 %; Control group: ↑25 % *CD4+* ^c^ Yoga group: ↓3.6 %; Control group: ↓3.5 % *CD8+* ^b^ Yoga group: ↓1.9 %; Control group: ↓3.7 % *CD56+* ^b^ Yoga group: 0.7 %; Control group: ↓4.3 % *Adverse events* none				
Vadiraja et al. (2009a)	*Intervention group* At least three in-person sessions/wk of a set of breathing exercises, pranayama and yogic relaxation techniques for 6 weeks during adjuvant RT in hospital and self-practice as homework on the remaining days. Audio taped instructions provided for home practice sessions *Control group* 15-min counselling sessions every 10 days for 6 weeks		Yoga group		Control group	
		*PANAS scale*				
		Positive affect^b^	27.8 ± 7.1		23.3 ± 8.3	
		Negative affect^b^	12.9 ± 10.39		21.8 ± 10.8	
		*EORTCQoL C30*				
		Physical function	73.2 ± 25.2		68.9 ± 30.1	
		Role function	79.8 ± 34.4		72.8 ± 39.9	
		Emotional function^b^	75.1 ± 21.1		59.2 ± 23.3	
		Cognitive function^c^	90.5 ± 15.8		80.7 ± 24.1	
		Social function	54.9 ± 23.9		49.9 ± 24.2	
		*Adverse events* none				
Vadiraja et al. (2009b)	Same as in Vadiraja et al. (2009a)			Yoga group	Control group	
		*RSCL*				
		Psychological distress^b^		4.2 ± 3.3	7.7 ± 3.4	
		Physical distress^c^		10.8 ± 8.1	15.0 ± 8.0	
		Activity level		20.2 ± 5.6	17.7 ± 6.2	
		*EORTCQoL C30*				
		Fatigue^b^		33.2 ± 23.8	50.5 ± 22.3	
		Pain^b^		24.4 ± 28.5	41.3 ± 28.9	
		Dyspnea		6.67 ± 15.2	9.8 ± 16.9	
		Insomnia^c^		24.4 ± 30.4	37.9 ± 31.7	
		Nausea and vomiting		9.6 ± 19.6	9.9 ± 17.3	
		Appetite loss^c^		17 ± 23.1	31.1 ± 28.1	
		Diarrhoea		0.7 ± 4.9	3.8 ± 12.8	
		Constipation		8.1 ± 23.7	9.1 ± 21.9	
		*Adverse events* none				
Vadiraja et al. (2009c)	Same as in Vadiraja et al. (2009a)			Yoga group	Control group	
		*Diurnal salivary cortisol*				
		6 a.m.^c^		0.22 ± 0.15	0.36 ± 0.24	
		9 a.m.		0.19 ± 0.14	0.24 ± 0.23	
		9 p.m.		0.16 ± 0.16	0.16 ± 0.14	
		*Hospital Anxiety Depression Scale*				
		HADS anxiety score^a^		4.8 ± 3.3	8.1 ± 3.8	
		HADS depression score^b^		4.1 ± 3.4	6.5 ± 3.7	
		Perceive stress scale^a^		15.1 ± 4.8	20.1 ± 5.8	
		*Adverse events* none				
Kumar et al. (2013)	*Intervention group* Participants were trained in Sudarshan Kriya and Pranayam through a 18-h contact program over a three-day period by trained yoga teachers. The program included teachings for self‑awareness Ujjayi breath, Bhastrika pranayama and rhythmic breathing. A 20-min home program was given for practice at home. In addition, they also received counselling and pain treatment as per WHO ladder of NSAID and morphine group of medicines *Control group* WHO ladder of NSAID and morphine group of medicines and counselling			Yoga group	Control group	
		Serum cortisol^b^ (ngm/l mean ± SE)		341.4 ± 51.4	549.2 ± 69.5	
		Pain perception		↓By three points in on 0–10 verbal scale of pain in the intervention group compared to control group		
Chakrabarty et al. (2013)	*Intervention group* 6-week hospital based program consisting of Sheethali, Brahmari and Nadisodhna Prayanama. Program duration of 18 min/session, twice a day × 5 days/week *Control group*: no intervention			Yoga group	Control group	
		Protein thiols^a^ µmol/l		271.2 ± 91.2	216.1 ± 62.8	
		Glutathione ^b^ mg/hHb (Median, IQR)		24.2; 18.3, 30.5	19.1; 18.0–24.6	
		*Adverse events* none				
*Physiotherapy based interventions*						
Chopra et al. (2006)	One supervised and two unsupervised 15–20 min sessions of inspiratory and expiratory manoeuvres and forced abdominal expiration techniques for 8–10 days			Pre training (mean)	Post training (mean)	
		Breath hold time (s)		31.4	44.5	
		Tidal volume (ml)		560	1160	
		*Adverse events* not reported				
Gautam et al. (2011)	Warm up with active ROM exercise for shoulder, PRE for upper limb muscle groups, exercise program hand-out with logbook, and telephonic monitoring once a week *PRE program* Intensity: start with 50–60 % of 10 RM and progress as tolerated Repetitions: 1 set of 8–10 repetitions, increase to 12–15 repetitions Sets: start with 1 set, progress to 2 sets of 12–15 repetitions before increasing weight by 5–10 % Frequency: 5 days/week		Pre-exercise		Post-exercise	
		SF 36—PCS^c^	41.2		46.3	
		SF 36—MCS^c^	38.5		48.3	
		Limb volume (ml)^a^	2306.3 ± 627.8		2183.4 ± 597.4	
		Limb circumference (cm)				
		MCP joints^c^	19.1 ± 1.4		19.0 ± 1.3	
		Wrist joint^a^	16.4 ± 2.9		16.2 ± 1.9	
		15 cm DLE^a^	22.1 ± 3.1		21.4 ± 2.8	
		10 cm PLE^a^	31.0 ± 4.3		30.0 ± 4.4	
		*Adverse events* not reported				
Kumar et al. (2013)	Mechanism based physical therapy consisting of educational, cognitive-behavioural therapy and physical therapy approaches		Pre-intervention		Post-intervention	
		BPI—Cancer pain^a^	75.25 ± 3.77		40.12 ± 4.08	
		EORTCQoL C30 global health status/QoL^a^	42.5 ± 9.1		68 ± 6.59	
		*Adverse events* not reported				
Samuel et al. (2013)	*Intervention group* 6-week brisk walking programme and active exercise programme for muscle groups of upper limb and lower limb. Intensity: RPE of 3–5/10; duration: 15–20 min Frequency: 5 days/week Progression as tolerated *Control group* Advised to remain as physically active as possible Home exercise program after study completion		Intervention group		Control group	
		6MWD^a^ Median change; (IQR)	20; (0, 46.)		−100.8; (−189, 53)	
		SF 36—PCS	No change		↓18 %	
		SF 36—MCS	↑11.7 %^c^		↓75.2 %^b^	
		*Adverse events* none				
*Speech therapy based interventions*						
Subbarao et al. (1991)	Group I: commenced planned ST 2–3 weeks post operatively Group II: received ST 1 yr after surgery Speech therapy which consisted of individual and group therapy sessions	↑improved proficiency in esophageal speech at various levels (Belch, Monosyllable, Bisyllable and simple sentences) in group I compared to group II *Adverse events* none				
Premalatha et al. (1997)	20–25 ST sessions of 30–40 min* 1–2 sessions/day ST included Inhalation technique, Inhalation combined with changing neck position and digital pressure.	↑Quality of voice and Speech intelligibility; better in patients using digital pressure. (No statistical analysis available) *Adverse events* None				
Bachher et al. (2002)	Supervised and home based ST to correct dyslalia and deglutition. 4 week supervised program: first 2 weeks @ 25–30 min/day; 3rd week @ 3 sessions/week; 4th week @ 2 sessions/week 3 months of home program: 15-min therapy sessions after every 1 h	Improvement in speech and deglutition (No statistical analysis available) *Adverse events* not reported				
John et al. (2011)	15 sessions of 30 min duration Program details: range of motion exercises, compensatory swallowing techniques and counselling on the altered swallowing manoeuvre	Improvement in swallowing, speech intelligibility, speech rate and articulation *Adverse events* not reported				

*BDI* Beck’s depression inventory, *BPI* brief pain inventory, *CINV* chemotherapy induced nausea and vomiting, *CT* chemotherapy, *DLE* distal to lateral epicondyle, *EORTCQoL C30* European Organisation for Research and Treatment of Cancer—quality of life C30, *FLIC* functional living index for cancer, *HADS* Hospital Anxiety and Depression Scale, *IQR* inter-quartile range, *MANE* morrow assessment of nausea and emesis, *MCP Joint* metacarpophalengial joint, *MCS* Mental Component Score, *NR* not reported, *NSAID* non-steroidal anti inflammatory drugs, *PANAS Scale* Positive and Negative Affect Schedule Scale, *PCS* Physical Component Score, *PLE* proximal to lateral epicondyle, *QoL* quality of life, *RSCL* rotterdam symptom check list, *RT* radiotherapy, *SF-36* short form (36) health survey, *STAI* state trait anxiety inventory, *TNF* tumor necrosis factor, *WHO* World Health Organisation, *6MWD* 6 min walk distance

^a^Significant at *p* < 0.001

^b^Significant at *p* < 0.01

^c^Significant at *p* < 0.05

## Discussion

The present study is the first comprehensive systematic review on exercise interventions for cancer survivors from India. Cancer survivorship is emerging as an important public health concern (Fairley et al. 2009), and exercise interventions are gaining importance as an integral component of supportive care therapies for cancer survivors (Jones and Demark-Wahnefried 2006). Studies addressing the role of exercise for supportive care in cancer has progressively increased since 1960s (Rigan 1963; Schmitz et al. 2005; McNeely et al. 2006; Sharma et al. 2013). Most of these studies have been conducted in high income countries. Despite having a National Cancer Control Program since 1975 (Ministry of Health and Family Welfare 2015), exercise interventions for cancer survivors has not received adequate attention in India from a policy perspective. In 2002, Ministry of Health and Family Welfare, Government of India, published a book highlighting the history, current state and future of cancer control in India (Agarwal et al. 2002). Though the book has a dedicated chapter on supportive care in oncology which addresses issues like cancer pain, nausea and vomiting, neutropenias, nutritional support, haematologic support, palliative care, and alternative medicine; exercise finds a mention only as a minor therapeutic option for pain management. Notwithstanding the lack of policies or guidelines on exercise interventions for cancer survivors in India, the current review highlights the emergence of research in this important aspect of cancer care from India.

Three distinct categories of exercise interventions were identified, of which, yoga was the most common (38.5 %). All five studies on yoga were conducted on breast cancer survivors and the outcome measures ranged from post-operative recovery period to anxiety, depression and quality of life (Raghavendra et al. 2007; Rao et al. 2008a, b; Vadiraja et al. 2009a, b, c; Chakrabarty et al. 2013; Kumar et al. 2013; Banerjee et al. 2007). The outcome measures reported in the five studies were diverse and precluded a meta-analysis. Though all five studies were RCTs, they had low to moderate methodological quality rating scores (range 14–19 out of 32) and most were not adequately powered to test the effect of interventions. Previous reviews have also highlighted high risk of bias or low methodological quality in among studies on yoga interventions (Sadja and Mills 2013; Shneerson et al. 2013).

The practice of yoga has a strong historical and cultural significance in India (Feuerstein 2013), which has emerged as one of the leading countries for studies on yoga interventions. A recent bibliometric analysis of yoga interventions identified 312 randomised trials of which 43.5 % were from India (Cramer et al. 2014). Yoga is currently gaining prominence as an alternative for improving physical and mental wellbeing even in western societies (Barnes et al. 2008). With many reviews and guidelines published within the last couple of years, evidence of its effectiveness in alleviating adverse effects of primary cancer therapy and in improving quality of life is emerging (Sadja and Mills 2013; Mustian 2013; Greenlee et al. 2014; Pan et al. 2015). However, a majority of studies on cancer survivors have been restricted to breast cancer. The bibliometric analysis of yoga interventions identified 17 studies in cancer and all were on breast cancer survivors (Cramer et al. 2014). With a prominent historical and cultural lineage towards yoga interventions, researchers in India have an opportunity to expand scope of yoga interventions to other forms of cancer. India has a significant burden of head and neck cancer and lung cancer and both types of cancer are known to have debilitating impact on survivors (Mathers et al 2012; Lewison and Roe 2012). Yoga interventions, with their focus on breathing control and on mind body integration, could be potentially useful in alleviating complications of primary cancer therapies among head and neck and lung cancer survivors. With Indian Prime Minister’s pitch for an International Day of Yoga and subsequent decision of the United Nations with support from 177 Nations to celebrate International Day of Yoga on 21st June (Ministry of Health and Family Welfare 2014), the environment is ripe for initiating high quality research on yoga interventions for survivors of various types of cancer.

In addition to yoga based exercise intervention, this review identified four physiotherapy based exercise interventions among cancer survivors (Gautam et al. 2011; Chopra et al. 2006; Samuel et al. 2013; Kumar et al. 2013). Among cancer survivors, the term ‘exercise intervention’ has conventionally been associated with interventions that focus on either or all of aerobic capacity, fitness, muscle strength and endurance, and physical activity; and are usually delivered at a given frequency and intensity. Although these interventions have been delivered by many healthcare professions (such as physiotherapist**s**, exercise physiologists, fitness specialists and nurses); the interventions come under the realm of physiotherapy practice and thus we classified them as physiotherapy based interventions. Among the four studies, only one was an RCT (Samuel et al. 2013), two were single group pre-post intervention design (Gautam et al. 2011; Chopra et al. 2006), and one was a case series (Kumar et al. 2013). The three studies that were eligible for methodological quality rating got a score of 16 (maximum score 32) (Gautam et al. 2011; Chopra et al. 2006; Samuel et al. 2013). A total of 115 survivors were studied across the four studies and reported on the effect of interventions on tidal volume, limb circumference and volume, pain, functional capacity and quality of life. Research in this category of exercise intervention is clearly lacking in India.

Compared to yoga based interventions, a greater body of evidence on effectiveness of physiotherapy based interventions is available. They have been shown to reduce recurrence of cancer, anxiety, depression, pain, and body mass index; and improve survival, cardiorespiratory fitness, strength, fatigue, range of motion, appetite, and quality of life (Fong et al. 2012; Jones and Demark-Wahnefried 2006; Schmitz et al. 2005; McNeely et al. 2006; Boughton 2006). The importance of these interventions has resulted in publication of guidelines to enhance and support evidence based clinical practice (Schmitz et al. 2010; Kushi et al. 2012; Wolin et al. 2012). As reasonable evidence exists on the effectiveness of physiotherapy based interventions, researchers in India could attempt to answer research questions related to relevance of existing guidelines in Indian context, effective ways to improve exercise prescription in routine clinical practice, and ways to improve exercise adherence and behaviour. Studies comparing or integrating yoga based interventions with traditional exercise interventions could lead to new approaches to exercise prescription for cancer survivors. Cancer survivors are known to be at increased risk of developing chronic diseases and are significantly more likely to die from non-cancer causes than the general population (Hewitt et al. 2003; Brown et al. 1993; Parkin et al. 2005). India currently has a high burden of chronic diseases and Indians are considered to have a lower threshold for developing chronic diseases. Relationship between exercise and risk factor profile for chronic disease among cancer survivors could also be a potential area for research.

The third category of exercise intervention identified through this review was based on speech therapy interventions. Though speech therapy may not be traditionally considered as exercise interventions, we included studies that used various exercises to improve speech and language, oromotor, and deglutition functions among cancer survivors. Survivors of head and neck cancer often face difficulties in speech and language, oromotor, and deglutition functions (Lam and Samman 2013; Wall et al. 2013; Patterson et al. 2015). Exercises have been shown to be effective in improving these functions and are recommended to be part of therapy even before initiation of chemo-radiotherapy (Paleri et al. 2014; Starmer 2014). Through this review, we identified only four studies from India, which consisted of one retrospective study (Subbarao et al. 1991), one case report (John et al. 2011), one case series (Premalatha et al. 1997), and one pre-post intervention study (Bachher et al. 2002). The studies reported data from a total of 92 head and neck survivors. Most studies were poorly reported with only one study qualifying for methodological quality rating (score of 5/32). These four studies indicated improvement in speech proficiency and intelligibility, voice quality, and deglutition.

Speech therapy based interventions among cancer survivors are most relevant to cancer of head and neck. India has a very large burden of head and neck cancer wherein it accounted for 29.8 % of all cancer in men and 10.6 % of all cancer in women (National Cancer Registry Programme 2013). Data from only 92 survivors is not commensurate with the burden of head and neck cancer in India. There is a strong need to study the prevalence of speech and language, oromotor, and deglutition related impairments, current clinical trends in exercise prescription and timing of initiation of exercise programs. High quality RCTs are needed to study the effect of exercise interventions for these impairments and also to study their effects on improving function and quality of life.

In addition to the categories of exercise interventions, this review also highlighted dearth of research with regard to cancer sites other than breast and head and neck; and various important outcome measures. Lung cancer is one of the leading sites of cancer in India and it was surprising to not find studies on exercise interventions among lung cancer survivors from India. In addition to breast, head and neck and lung cancer, current literature indicates emergence of evidence on benefits of exercise among survivors of prostate, colon, hematologic and gynaecologic cancer (Schmitz et al. 2010). Research from India could add to the body of knowledge on benefits of exercise interventions for various cancer sites and help strengthen global recommendations for exercise prescription. Effect of exercise interventions on hard outcomes measures like cancer recurrence and survival have not been addressed through research from India. Evidence of reduced recurrence rates and improved survival could put the spotlight on exercise interventions in India.

The current review indicates that research on exercise based interventions among cancer survivors is at a nascent stage in India. Several factors could be attributed for the lack of research in this area. India’s cancer control program had an initial focus on providing cancer treatment facilities and later modified to include prevention and early detection on cancer. Exercise interventions, as of now, do not seem to be a priority area under the National Cancer Control Program. A lack of priority at a policy level could have led to reduced awareness about role of exercise interventions for survivors among physicians and allied health professionals. Lack of exercise professionals trained to cater to the needs of cancer survivors could also be a reason for limited research in this area.

It is encouraging to notice a consistent improvement in exercise based research despite absence of policy on exercise interventions for cancer survivors in India. From a mere two studies during the 1991–2000 decade, the number of studies have increased to five during the 2000–2009 decade and six studies have already been published within 4 years of the current decade. Exercise based research for cancer survivors has a long way to go in India. There is a need to sensitize and train oncologists, exercise professionals, nurses and other healthcare providers about the benefits of exercise intervention for cancer survivors. There is also a need for grants and programs that will train professionals from India to administer exercise based interventions as a routine part of treatment and management for cancer. It may be beneficial to consider including supportive cancer therapies including exercise based interventions as part of National Cancer Control Program.

This review, by highlighting the state of research in exercise based interventions among cancer survivors in India, opens up the possibility for researchers from high income countries to identify researchers in India and initiate high quality multinational clinical trials on exercise interventions. India, with its strength on Yoga based interventions, could offer a unique opportunity for researchers from other countries to collaborate and test yoga based interventions in different populations and cultures.

### Strengths

To the best of our knowledge, this is the first systematic review highlighting the state of exercise based interventions for cancer survivors in India. For the purpose of this review, we used a broader definition of exercise to include all forms and categories of exercise interventions. This resulted in identifying yoga based interventions and speech therapy-based interventions from India. We used a comprehensive set of search terms developed through a consensus process among subject experts and covered most important databases including India specific databases like IndMed and Shodhganga. Such a strategy is likely to have greater sensitivity and could be considered reflective of state of exercise based research in India. We also used a validated methodological quality checklist that helped evaluate the strengths and weakness of studies included in the review.

### Limitations

Though we began the review with an intention to perform meta-analysis, the variability in study characteristics and outcomes precluded the scope of conducting a meta-analysis. Though we screened for doctoral level theses from India through Shodhganga, the normal delays in uploading the thesis to the central repository by many Universities could have resulted in us missing out on doctoral level theses that have not been published in peer-reviewed indexed journals. There is also a possibility of postgraduate dissertation research on exercise based interventions that are currently not accessible due to lack of a comprehensive database of postgraduates dissertations from India.

## Conclusion

The present review indicates a paucity of research examining exercise-based interventions among cancer survivors within the socio-cultural context of India. Though a positive trend in findings was observed, future research, of higher quality and including a broader range of cancer types and outcomes, is urgently needed.
